# Open Targets: a platform for therapeutic target identification and validation

**DOI:** 10.1093/nar/gkw1055

**Published:** 2016-12-08

**Authors:** Gautier Koscielny, Peter An, Denise Carvalho-Silva, Jennifer A. Cham, Luca Fumis, Rippa Gasparyan, Samiul Hasan, Nikiforos Karamanis, Michael Maguire, Eliseo Papa, Andrea Pierleoni, Miguel Pignatelli, Theo Platt, Francis Rowland, Priyanka Wankar, A. Patrícia Bento, Tony Burdett, Antonio Fabregat, Simon Forbes, Anna Gaulton, Cristina Yenyxe Gonzalez, Henning Hermjakob, Anne Hersey, Steven Jupe, Şenay Kafkas, Maria Keays, Catherine Leroy, Francisco-Javier Lopez, Maria Paula Magarinos, James Malone, Johanna McEntyre, Alfonso Munoz-Pomer Fuentes, Claire O'Donovan, Irene Papatheodorou, Helen Parkinson, Barbara Palka, Justin Paschall, Robert Petryszak, Naruemon Pratanwanich, Sirarat Sarntivijal, Gary Saunders, Konstantinos Sidiropoulos, Thomas Smith, Zbyslaw Sondka, Oliver Stegle, Y. Amy Tang, Edward Turner, Brendan Vaughan, Olga Vrousgou, Xavier Watkins, Maria-Jesus Martin, Philippe Sanseau, Jessica Vamathevan, Ewan Birney, Jeffrey Barrett, Ian Dunham

**Affiliations:** 1 Open Targets, Wellcome Genome Campus, Hinxton, Cambridge, CB10 1SD, UK; 2 GSK, Medicines Research Center, Gunnels Wood Road, Stevenage, SG1 2NY, UK; 3 Biogen, Cambridge, MA 02142, USA; 4 European Bioinformatics Institute (EMBL-EBI), Wellcome Genome Campus, Hinxton, Cambridge, CB10 1SD, UK; 5 Wellcome Trust Sanger Institute, Wellcome Genome Campus, Hinxton, Cambridge, CB10 1SA, UK; 6 National Center for Protein Research, No. 38, Life Science Park Road, Changping District, 102206 Beijing, China

## Abstract

We have designed and developed a data integration and visualization platform that provides evidence about the association of known and potential drug targets with diseases. The platform is designed to support identification and prioritization of biological targets for follow-up. Each drug target is linked to a disease using integrated genome-wide data from a broad range of data sources. The platform provides either a target-centric workflow to identify diseases that may be associated with a specific target, or a disease-centric workflow to identify targets that may be associated with a specific disease. Users can easily transition between these target- and disease-centric workflows. The Open Targets Validation Platform is accessible at https://www.targetvalidation.org.

## INTRODUCTION

The fundamental tenet of pharmacology is that a drug (small molecule or biological) can be identified that specifically interacts with a target molecule (usually a protein) to modulate a physiological process and thus alter the course of a disease (1,2). The pharmaceutical industry has developed powerful approaches to discover and optimize drug molecules that affect the function of a target. There are also complex strategies in practice to deal with drug efficacy, dosing and safety issues that accompany getting a drug into humans and finally to market. However, analysis of progress through development pipelines has highlighted that lack of efficacy is a major cause of failure, particularly in the later, more expensive, clinical stages (3,4). The implication is that the link between the target and its influence on physiology and disease was not well enough established, and that better assessment of the evidence behind the role of the target in disease might improve success rates and/or allow early termination of implausible development programs (5).

Historically, drug targets have been chosen on the basis of the accumulation of a series of experimental observations that support the hypothesis that modulating the function of the protein will have an effect on disease. The staggering improvements in high throughput technologies such as nucleic acid sequencing, genotyping and mass spectrometry of metabolites or proteins are allowing detailed characterization of biological samples, and have opened up new sources for discovery of disease biology. Several recent publications have championed the value of genetic information from genome-wide association studies (GWAS) and Mendelian inheritance in the identification and prioritization of potential targets (6–9). Indeed drug development programs that have supporting genetic information are more likely to proceed into the final stages (3,10). The growing volume of genetic information can be a rich source for target identification, while the other high throughput methods can provide extensive additional supporting information. Furthermore recent developments in gene editing that allow direct manipulation of the genome of somatic cells (11,12) promise to provide data on target modulation in human cells to supplement the results from more established technologies in model organisms.

In this context, we (Biogen, EMBL European Bioinformatics Institute, GlaxoSmithKline and the Wellcome Trust Sanger Institute) have come together to form Open Targets (http://www.opentargets.org), a public-private partnership to establish an informatics platform, the Target Validation Platform. Its aim is to provide comprehensive and up to date data including but not limited to relevant genetics and high throughput genomics data for drug target selection and validation. Here we describe that platform, and the approach we used to develop it.

## THE OPEN TARGETS TARGET VALIDATION PLATFORM

### Linking targets to disease via evidence found in public data sources

The Target Validation Platform is available at https://www.targetvalidation.org. It allows investigation of the evidence that associates targets and diseases in an intuitive and accessible manner, while providing tools to prioritize these target-disease hypotheses for further follow-up. The evidence that is integrated into the platform comes from public domain data sources and includes rare and common disease genetics, somatic mutations in cancer, transcriptomics, approved drugs and clinical candidates, animal models, biochemical pathways and text mining from the medical literature.

**Figure 1.**
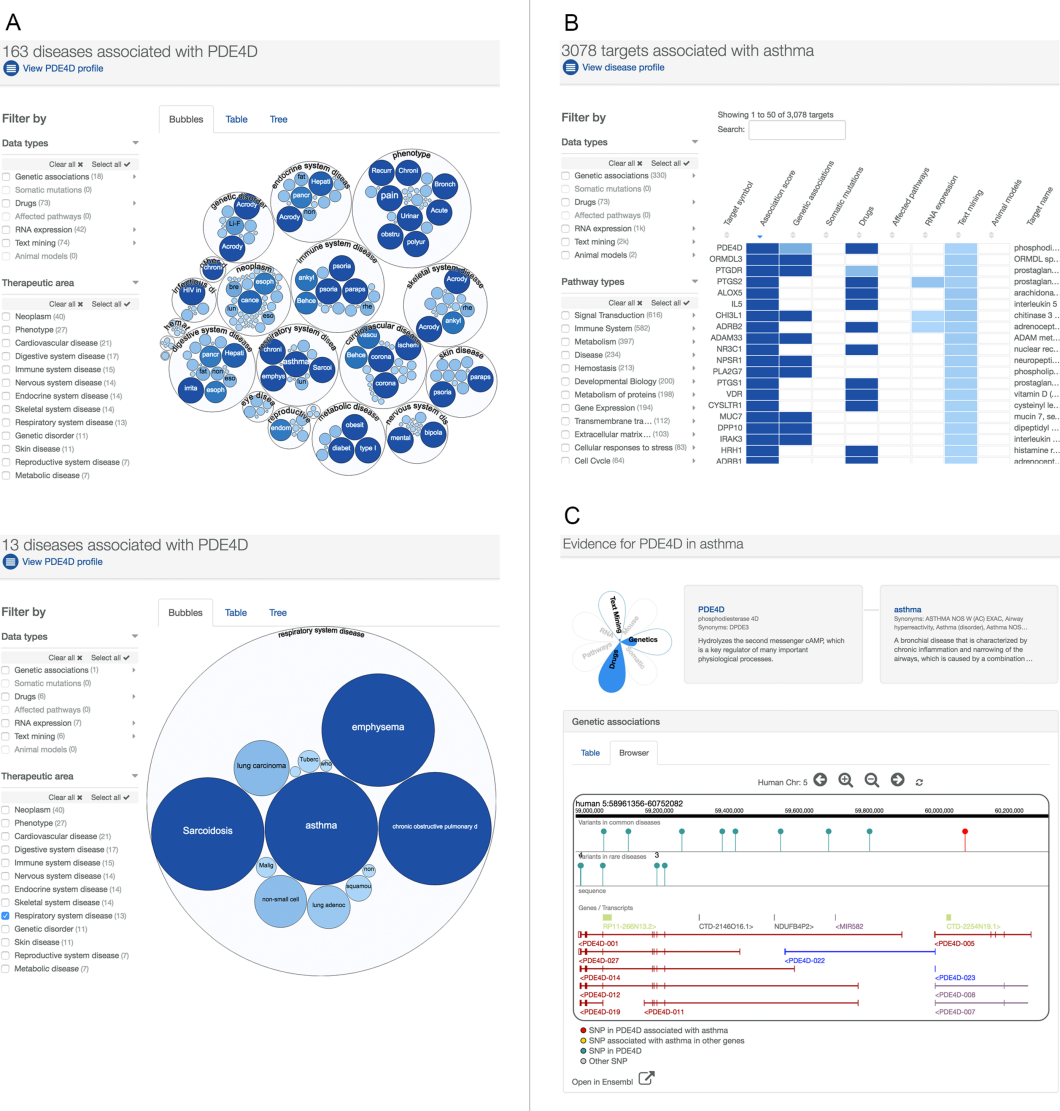
Workflows of the application. (**A**) Platform workflow for PDE4D: the user has searched for the gene name ‘PDE4D’ and is presented with all the diseases associated with this gene including asthma. Diseases are presented as ‘bubbles’ grouped into therapeutic areas using their EFO relationships. The size and shade of blue color of each bubble is proportional to the strength of association between the disease and PDE4D. Diseases can also be presented as a table or a tree (based on the EFO (17)) by clicking on the corresponding tabs. Selecting the ‘Respiratory system disease’ filter, displays the diseases in this therapeutic area which are associated with PDE4D. (**B**) Platform workflow for asthma: conversely, the user can enter the disease term ‘asthma’ and will be presented with all the associated targets for asthma including PDE4D. (**C**) Evidence for PDE4D in asthma: Clicking on asthma in panel A or on PDE4D in panel B shows the types of evidence which support the association between PDE4D and asthma. The evidence ‘flower’ provides an overview of the strength of the association for each type of evidence. Details of the available evidence are presented as summary tables or graphical displays. For example, the red pin in the gene browser below shows the position of a SNP in PDE4D associated with asthma.

The application supports two main workflows (Figure 1). First, the user can enter a target and will be presented with visualizations of the evidence for associations with specific diseases grouped by broad therapeutic areas. Further pages allow in-depth examination of the evidence and user-defined prioritization of the lists of associations. Second, the user can enter the name of a disease to ask which targets may be associated with this disease. This leads to pages that summarize the targets linked with that disease and the underlying evidence. For instance, in Figure 1, the user can enter a gene name or gene symbol like ‘PDE4D’ and retrieve all the associated diseases including asthma. Conversely, the user can enter the disease term ‘asthma’ and retrieve the associated targets for asthma including PDE4D. Further pages provide profiles of the targets and diseases, and graphical and textual displays of evidence for associations and basic biological data. We will return to describe the benefits of the two workflows later, but first we explain the motivation for our approach and the technical implementation.

### User experience design methods helped us understand the needs of potential users to design an intuitive target validation platform

Through the Target Validation Platform we aim to empower practicing biological scientists in the pharmaceutical industry and in academia to select and prioritize the targets most likely to succeed based on data driven associations with diseases. No in-depth understanding of bioinformatics or the integrated data should be required for them to make use of the platform.

To achieve this goal, we applied a range of User Experience (UX) design methods (13–15). At the beginning of the project, we interviewed scientists and managers working in pharmaceutical research and development, as well as academic researchers interested in drug discovery. We discovered the questions they ask in order to identify and prioritize targets, and the paths they take toward validation. This helped us to understand the ecosystem of data that drug discovery practitioners use to build early confidence in a target.

We synthesized the information that we had collected from the users and identified initial overarching questions that we would be required to answer via the platform. Primarily, our users were interested in finding out:

- Starting from a particular target (e.g. PDE4D), which diseases are associated with the target?
- Starting from a particular disease (e.g. asthma), which targets are associated with this disease?

The two workflows in Figure 1 were designed to provide answers to these questions based on the evidence that is integrated in the platform.

A key message from the interviewees was the requirement to assemble as much as possible of the relevant evidence that a target was involved in a disease in a *single place*, and to assign *priority* (scores) to the target-disease association in the correct biological context (such as tissue, organ, etc.). This evidence is often dispersed and not easily accessible to researchers in drug discovery without the support of specialized data scientists (bioinformaticians and computational biologists). Therefore, our main objective for the platform was to provide these researchers with more direct and user-friendly access to that evidence.

To further understand which data is more valuable for our audience and to develop appropriate ways to display these data, we ran collaborative design workshops. These helped us to formulate initial designs for the platform, which we showed to other potential users for additional feedback.

We iteratively improved the designs for the platform based on user feedback first using sketches, then wireframes and later using interactive prototypes. To achieve this we recruited around 100 beta testers who provided feedback on the platform at various stages of development and made sure that it met their expectations and was appropriate for the tasks we wished to support.

The UX design process that we followed is discussed in more detail elsewhere (16).

### The data model stores relevant evidence that associates a target with a disease

The Target Validation Platform stores relevant evidence that associates a target with a disease for all potential human targets including proteins and RNA molecules. However we do not wish to store all the data contributing to the evidence, partly for efficiency, but primarily because databases already exist that are uniquely tuned to deal with many of the specialized data sources and we fully expect these data sources to evolve in the future with future techniques. Instead we have chosen to develop summaries of the data encapsulated in the form of an evidence object that is either supplied by the source database, or prepared by us through an analytical pipeline or by parsing other databases. Thus we effectively have a federated structure with our core database providing summary information of the detail in the source databases.

**Figure 2.**
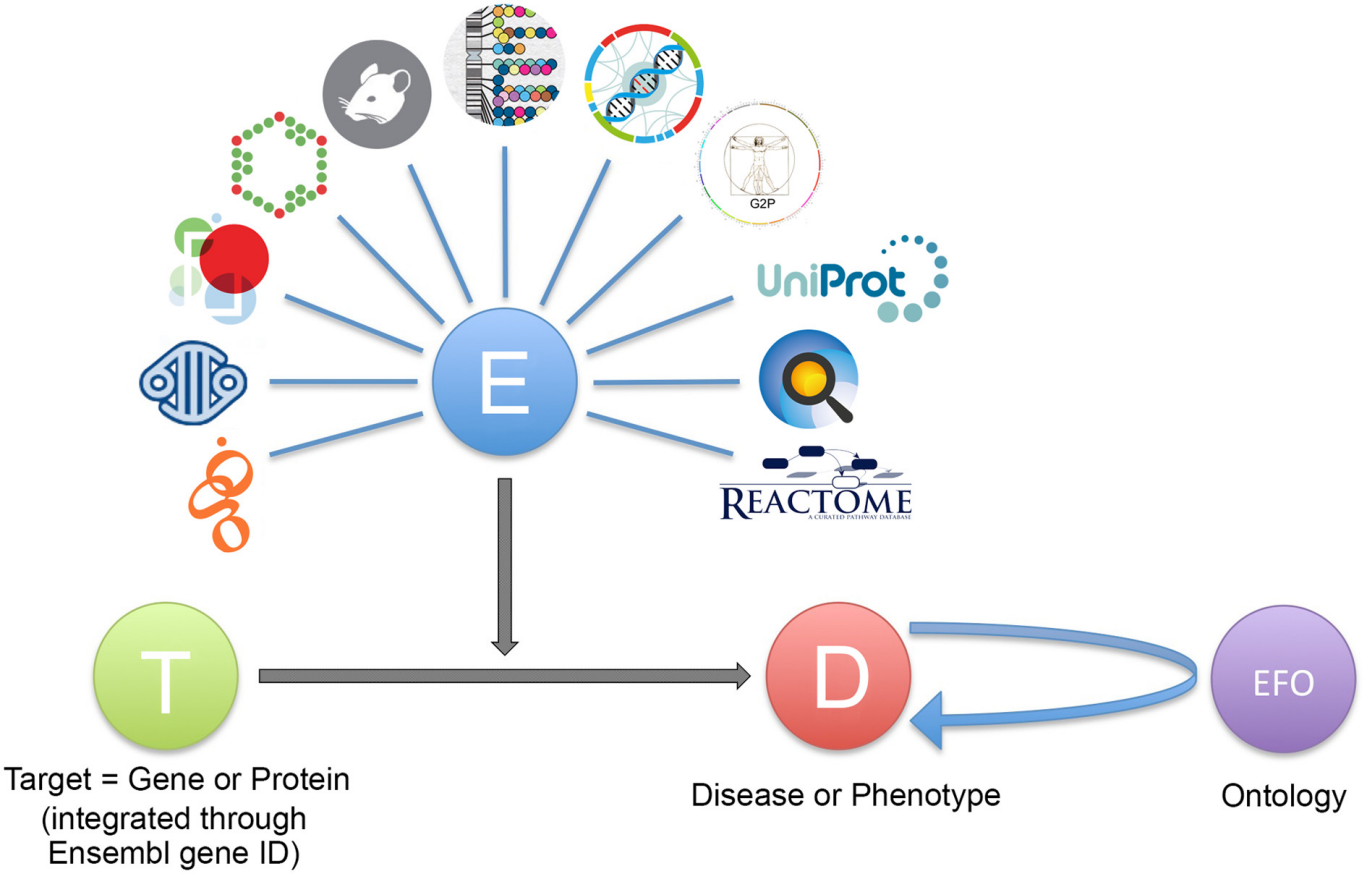
Schematic representation of the Open Targets evidence object associating a Target (T) with a disease (D). Evidence (E) is provided as JSON objects according to a JSON schema. Database icons are clockwise from bottom left IntOGen (32), Cancer Gene Census (31), Europe PMC (21), ChEMBL (49), PhenoDigm (50), GWAS Catalog (51), European Variation Archive (http://www.ebi.ac.uk/eva/), Gene2Phenotype (52), UniProt (23), Expression Atlas (53) and Reactome (47). Specific ontology terms have been defined to describe evidence and are part of the Open Targets evidence ontology, included in the experimental factor ontology (EFO).

To allow this design we have developed the concept of a target-disease association object that aims to capture and summarize the available information linking a target to a disease for a given experiment or database resource. The target can be a gene, transcript or protein (or indeed in principle any biomolecule) defined by standard nomenclature, while the disease is described by ontology terms from the experimental factor ontology (EFO) (17). The evidence is described in the Open Biomedical AssociatioN (OBAN) representation (18) and makes use of the Evidence Code Ontology (ECO) (19) (Figure 2) that in turn is part of EFO. For example, a similar approach has been used in nanopublications (20). In the current implementation, the evidence is provided as a JavaScript Object Notation (JSON, http://json.org) format object with a JSON schema (http://json-schema.org) that enables sufficient information about the evidence to be transmitted from the source database to the core. Each JSON document contains unique resource identifiers (URI) for a target and a disease, a list of Europe PMC (21) scientific literature references (which includes references to PubMed abstracts) when applicable, a provenance type (to describe the datasource), an ECO evidence code (e.g. computational prediction, curator inference) and the evidence linking the target to the disease. For instance, the association of a gene target with a disease through a genome wide association study (GWAS) is described by an evidence object including the GWAS association of a sequence variant (SNP) to a disease. The object also contains a description of the statistical method used, the reported p-value and the case versus control sample size, and the assignment of the SNP to a gene ideally via additional experiments such as eQTL mapping or chromatin interaction mapping. Each part of the evidence can have a score, allowing fine-grained modulation of an overall score for the association. This high level simplification allows different data to be handled in a uniform manner, and the flexibility of the data model representation makes it possible to create additional data types in the future with as little maintenance as possible. The Open Targets platform currently covers genetic associations, somatic mutations, know drugs, gene expression, affected pathways, literature mining and animal models (see Table 3).

A key requirement is that both target and disease must be referred to via the same terms in each of the source databases. The relationship between genes, proteins and other biomolecules is complicated both by biological processes such as alternative splicing and post-translational modification and by nomenclature issues. Fortunately, there are widely accepted solutions and mapping available for this problem, and we use Ensembl (22) identifiers on their reference genome build (GRCh38) as our key gene identifier, associated to UniProt (23) identifiers. However consistent description of disease and phenotypes is harder and has numerous differing standards. We have chosen to standardize on the EFO to allow us to traverse disease space, retrieve disease synonyms and definitions, and to compute relationships. To enable this we have started mapping several existing ontologies like ORDO (Orphanet Rare Disease Ontology (24)), DO (disease ontology, (25)) and HPO (Human Phenotype Ontology,(26)) or widely-used terminologies like MeSH (http://www.ncbi.nlm.nih.gov/mesh) and OMIM (27) to EFO, and we incorporate new human disease terms as necessary. This is achieved by including existing ontology concepts or by cross-referencing external concepts as described in (18). In the platform, we separate human diseases from phenotypes and we use existing HPO or MP (Mammalian Phenotype, (28)) terms to represent phenotypes. In addition we are using text mining and expert review to enhance the EFO coverage of diseases in our initial focus areas starting with Inflammatory Bowel Disease (IBD) and immuno-inflammation. As of October 2016, we have added 3663 new EFO classes and 31 332 axioms.

### Providing the evidence objects and scoring the associations

From engaging with users, we defined a set of core data types that were of primary importance to target validation. These include information on genetic associations with either common disease primarily from GWAS or rare Mendelian disease from sequencing of exons of protein coding genes; somatic mutations implicated in cancer; significant changes in gene expression in appropriate sample comparisons from microarray or RNA-seq experiments; existing drugs that engage a target and treat a disease; animal models with gene knockouts and phenotypes concordant with human disease; biochemical pathways that are affected by disease; and associations of targets with disease in the biomedical literature identified through text mining. We established data pipelines from existing world-recognized databases (Table 1) to provide evidence objects in each of these areas. Although each of the source databases contained relevant data, their primary focus was not usually on target validation so the standardization on the EFO within the databases and generation of additional ontology terms has had an additional integrating effect across these resources. Each of the data pipelines provides a set of target-disease evidence JSON objects with sufficient information to describe the evidence, and to calculate an association score. In addition, data is provided to enable URLs that link back to the data sources to be computed.

**Table 1.** Target-disease association objects available from the databases in the data pipelines

Database	Evidence type	Evidence objects
GWAS Catalog (51) (http://www.ebi.ac.uk/gwas/)	GWAS associations by lead SNPs	32 363
European Variation Archive (http://www.ebi.ac.uk/eva/)	Variants with clinical phenotypes	28 050
UniProt (23) (http://www.uniprot.org)	Proteins with known disease roles	21 870
Gene2Phenotype (52) (http://www.ebi.ac.uk/gene2phenotype/)	Genetic diagnosis of developmental disorders	975
Expression Atlas (53) (https://www.ebi.ac.uk/gxa/home)	Up/down regulated genes in disease	529 084
Cancer Gene Census (31) (http://cancer.sanger.ac.uk/cancergenome/projects/census/)	Cancer driver genes	23 440
IntOGen (32) (https://www.intogen.org/)	Cancer driver genes	2377
ChEMBL (49) (https://www.ebi.ac.uk/chembl/)	Approved and marketed drugs	120 520
Reactome (47) (http://www.reactome.org)	Reaction pathways altered by variant in disease	6143
PhenoDigm (50) (http://www.sanger.ac.uk/resources/databases/phenodigm/)	Animal model annotations	395 622
Europe PMC (21) (https://europepmc.org/)	Literature-based evidence	3 678 967

A key challenge is to integrate the data with joint computation of the likelihood that a target will affect disease across all the information. We have developed a scoring scheme to describe the overall confidence and strength of a target-disease association taking into consideration all the evidence available from many data types.

We derive an association score per evidence, per data source, per data type and overall. First, an association score ranging from 0 to 1 is derived for each piece of evidence (see Table 2). The calculation of the evidence association score follows a general framework that currently combines up to three component variables:

- Frequency representing the relative occurrence of a particular target-disease evidence,
- Severity expressing the magnitude or strength of the effect described by the evidence and
- Confidence assigning an overall confidence for the observation that generates the target-disease evidence.

**Table 2.** Database and scoring

Database	Evidence score
GWAS Catalog	*N*(pvalue) × *N*(sample size) × functional consequence score
European Variation Archive germline variants	Functional consequence score
UniProt	Curator inference score:
	A. 0.5 when there is not a strong evidence for the gene's involvement in the disease.
	B. 1 when there is a strong evidence for the gene's involvement in the disease.
Gene2Phenotype	Curator inference score = 1
Expression Atlas	*N*(pvalue) × *N*(expression fold change) × *N*(percentile rank)
Cancer Gene Census	Functional consequence score
European Variation Archive somatic variants	Functional consequence score
IntOGen	Binned score based on tumor type category:
	A. 0.25 if the gene exhibits several signals of positive selection in the tumor type
	B. 0.5 if the gene is already described as a cancer gene and exhibits a signal of positive selection in the tumor type
	C. 0.75 if the gene exhibits a signal of positive selection and is functionally connected to the genes with evidence A or B in the tumor type
ChEMBL	Clinical trial phase binned score:
	Phase 0: 0.09
	Phase 1: 0.1
	Phase 2: 0.2
	Phase 3: 0.7
	Phase 4: 1.0
Reactome	Curator inference score = 1
PhenoDigm	Original similarity score described in (50). The OWLSim algorithm determines the pairwise phenotype similarity of a mouse model and a human disease.
Europe PMC	Original confidence score described in (34) based on weighting document sections, sentence locations and title for full text articles and abstracts.

All individual evidence score range from 0 to 1. A function *N* is applied to normalize components of the score. Functional consequence scores can be found in the Supplementary Table S2 and UniProt curator inference rules in Supplementary Table S3.

For instance, for GWAS genetic evidence, the frequency is based on the sample size (case versus control), the severity represents the predicted functional consequence of the variation and the confidence corresponds to the *P*-value reported in the GWAS study. From a set of individual scored evidence objects a data source association score, *S*, is derived using a harmonic sum function (29,30) to account for replication but also to dampen the effect of large amounts of data such as obtained from text mining by calculating:

\begin{equation*}{S_{1..i}} = {S_1} + \frac{{{S_2}}}{{{2^2}}} + \frac{{{S_3}}}{{{3^2}}} + \frac{{{S_4}}}{{{4^2}}} \ldots . + \frac{{{S_i}}}{{{i^2}}}\end{equation*}

where, *S_1_, S_2_,…,S_i_* are the individual sorted evidence scores in descending order.

The same approach is applied to compute a data type score where similar data sources are grouped for scoring (see Table 3) and to derive an overall score for each target-disease association. For example, to compute the somatic mutation data type score for the association ‘BRCA2-breast carcinoma’, the data source scores from Cancer Gene Census (31), the European Variation Archive (http://www.ebi.ac.uk/eva/) and IntOGen (32) are sorted and the harmonic sum calculated. The overall score for the association ‘BRCA2-breast carcinoma’ combines all the data type scores (somatic mutations, text mining, etc.). Similarly, the overall score for the ‘PDE4D-Asthma’ association combines the genetic association data type score, the drug data type score and the text mining data type score.

**Table 3.** List of the Open Targets platform data types along with the list of their data sources

Data type	Data sources
Genetic associations	GWAS Catalog, UniProt, European Variation Archive, Gene2Phenotype
Somatic mutations	Cancer Gene Census, European Variation Archive somatic, IntOGen
RNA expression	Expression Atlas
Drugs	ChEMBL
Affected pathways	Reactome
Text mining	Europe PMC
Animal models	PhenoDigm

Some data sources provide their own scores that we reuse. For instance, we use the score provided by PhenoDigm for the relevance of a mouse model to a human disease. We also use the association score for literature-based evidence developed by Europe PMC for the purposes of Open Targets. The Europe PMC database (https://europepmc.org/) which covers >30.4 million abstracts and >3.3 million full text articles from PubMed and PubMed Central (21) is mined to identify associations between target and human disease. Two comprehensive resources, UniProt and the EFO are utilized to annotate target and disease names in text and extract the associations between them using an extension of Whatizit (33) and target-disease co-occurrences at the sentence level are extracted. Several heuristic filtering rules based on a careful manual analysis of the text data are applied to remove potential false positive associations. These rules include: (i) filtering out article types except ‘Research’ articles (e.g. Reviews, Case Reports), (ii) removing target-disease associations appearing in the Methods, References, Acknowledgement and Funding, Competing Interests, Author Contribution and Supplementary sections and (iii) filtering out target-disease associations that appear only once in the body of a given article but not in the article title or abstract. Document confidence scores for a given target-disease association are calculated by assigning different weights to the paper sections (such as Title, Abstract, Results, etc.) and are used in the target validation platform to rank all the documents relevant to a given target-disease association (34).

In addition, we use a weighting factor to adjust scores to control the relative contribution of the data source to the overall association score. For instance, we upweight the GWAS association score and downweight the text mining data source association score. Table 2 summarizes the parameters used from each data source to compute the individual evidence score.

## RELATED WORK

Several resources such as DrugBank (35), the Therapeutic Target Database (TTD,(36)), STITCH (37), PharmaGKB (38), SuperTarget (39) have been developed to provide comprehensive information on drug targets by integrating information from multiple sources. The emphasis of these databases is on the known and predicted interactions between the clinical trial drugs and their targets, how drug effects on targets are propagated through their corresponding pathways, their relationships to diseases, adverse events of drugs and pharmacogenomics, rather than the evidence associating targets and disease.

More recently, the NIH has launched the Illuminating the Druggable Genome (IDG; https://commonfund.nih.gov/idg/) program to find potential new drug targets within the four most commonly drug-targeted protein families (G-protein coupled receptors, nuclear receptors, ion channels and protein kinases). Two discovery platforms are developed as part of this program: the Harmonizome (40), a comprehensive resource of knowledge about genes and proteins and PHAROS (https://pharos.nih.gov/) which follows a similar approach to Open Targets by integrating multiple sources of biomedical data, albeit concentrating on four protein families.

DisGeNET (41) is the closest resource to the Open Targets Target Validation Platform in terms of information on Mendelian and complex diseases to help prioritization of disease genes as targets. It builds a data model on gene-disease associations and applies ontology standards to define diseases and phenotypes. Both platforms integrate information from curated or predicted biomedical data sources and from the literature. A gene-disease association score is generated to rank the associations on the supporting evidence. While both of the resources provides curated genomic information from UniProt, ClinVar and the GWAS Catalog, a notable difference is that the Open Targets target validation platform provides additional target-disease association through approved drugs and clinical candidates, RNA expression and biological pathways disrupted by genetic mutations information that are not available in DisGeNET. However, DisGeNET provides additional gene-disease association via the Comparative Toxicogenomics Datatabase (CTD, (42)) to cover the effects of environmental chemicals on human health. These differences reflect differences of focus of the two tools. The difference in evidence coverage is reflected in the relative ranking of targets associated with diseases in the two systems. For instance, for Alzheimer's disease, APP and SORL1 are ranked highly in both while PSEN1, PSEN2, CLU and APOE are ranked differently due to the additional clinical trial drug information contained in the Open Targets Platform.

## IMPLEMENTATION

### Implementing the integration in the target validation platform

All the data from the external data sources are stored as JSON documents in ElasticSearch, a distributed, highly-performant and scalable full-text search engine based on Lucene (https://www.elastic.co/). This includes the original evidence and any other biological information (e.g. gene and protein identifiers and synonyms, GO terms (43), UniProt information, Reactome pathways), biomedical ontologies (e.g. EFO, ECO, GO) or functional consequence terms from SO (44) required to integrate and serve the data. We have built a data pipeline to handle and process the different target-disease evidence sources. The input JSON data is validated to check its format, current biological identifiers and ontology references. This includes checking the validity of the gene or protein information against the current version of Ensembl and UniProt, and verifying that the disease and phenotype information exists in the latest version of EFO or is a genuine HPO/MP term. The following steps of the pipeline analyze all the available evidence, compute their individual evidence scores and combine them to derive higher order target-disease association objects and scores. To date (release 1.2) we have derived 2 484 000 association objects from 4 840 000 evidence (Table 1 and Supplementary Table S4) covering 30 591 targets and 9425 diseases and phenotypes. Indeed, by using the EFO parent-child (subclass of) relationships, we derive new associations that may not have direct evidence. For instance, IBD is an autoimmune disease and the direct evidence of targets associated to IBD are propagated to the higher autoimmune level to allow users to find common targets across groups of related diseases (e.g. Ulcerative Colitis, Crohn's disease and IBD). In EFO, ‘asthma’ is a ‘respiratory system disease’, ‘childhood onset asthma’ is a subclass of ‘asthma’. Consequently, both evidence from ‘asthma’ and ‘childhood onset asthma’ are propagated to ‘respiratory system disease’. Other relations can be derived based on EFO inferred-by-property classification: disease location (e.g. respiratory system, endocrine system), disease cell type (epithelial cell in epithelial neplasms) or cell lines (lung cancer cell lines) and disease phenotypes (e.g. thrombosis in ulcerative colitis). This will enable us to group related diseases based on these properties. We provide access to common disease genetic evidence based on GWAS study results from the GWAS Catalog and rare Mendelian disease evidence based on clinical variant information accessible from EVA. We developed a pipeline to systematically assign genomic sequence variants from the GWAS catalog to protein-coding genes. Inter-genic SNPs are assigned to the nearest gene five prime end. Deleterious effects of variants on transcripts were annotated with SO terms using the Ensembl Variant Effect Predictor (VEP) (45). For instance, the NOD2 variant p.Leu1007fsX1008 (rs2066847) is associated with Crohn's disease, a chronic inflammatory disorder of the gastrointestinal tract (46). The pipeline mapped rs2066847 to NOD2 in exon 11 and annotated it as a frameshift variant (SO:0001589).

### Implementation of intuitive data visualizations

The evidence visualizations used in the platform have been developed as reusable Javascript components, meaning that they can be seamlessly reused in other host web applications to display Open Targets data. Some of them, including a lightweight genome browser and a phylogenetic tree visualization module are registered in BioJS (https://biojs.net/), a registry of Javascript modules to represent biological data.

To visualize data related to targets or diseases but outside our core data concerning the associations between targets and disease, we integrate third party visualizations developed by the community. These ‘widgets’ include a visualization for biological pathways developed by Reactome (47), a graphical display of RNA baseline expression developed by Expression Atlas (53), a visualization of the different protein features developed by UniProt (23) or a three-dimensional protein structure display for targets (http://dx.doi.org/10.5281/zenodo.20980). In addition, the web platform has been designed to incorporate other third party widgets to visualize target or disease information in any local or user-deployed instance.

### Public access to data and code repository

The platform is available at https://www.targetvalidation.org. We offer an open access to data directly through an application programming interface (API) and via bulk downloads. Documentation on how to use the API and the supported methods is available on the API documentation page (https://www.targetvalidation.org/documentation/api). To facilitate programmatic access to the API the Open Targets team supports three clients written in Javascript, Python and R, respectively. The source code of these clients, the evidence JSON schema, the python validation package and the web application code are available on GitHub (https://www.github.com/CTTV).

### The target validation platform helps researchers

Part of our UX process was to define key metrics to assess the Target Validation Platform. We did this based on the HEART methodology (48), focussing on Adoption, Engagement and Retention as the main aspects of UX for the platform. Supplementary Table S1 reports the averages of these metrics for the 24 weeks from 29 February until 2 October 2016.

Overall, our analytics suggest that the Target Validation Platform is being used substantially by the target audience (Supplementary Table S1). This is aligned with the qualitative feedback we have been receiving from users. As one drug discover researcher said: ‘Powerful resource, clear links and easy to use without training, especially for a non-bioinformatician!’. We continue to collect feedback on the various aspects of the platform and we will be introducing new features following the same iterative UX design process.

## CONCLUSION

Our use of UX design methods place the eventual user of the Target Validation platform at the center of design and development decisions to produce a platform (https://www.targetvalidation.org) which delivers integration and answers to key target selection and validation questions asked at the start of the drug discovery process. We will continue to develop the platform to provide additional data, and further methods for prioritizing targets including exploiting tissue and cell specific expression patterns. Further work will include enriching the disease ontology, refining the scoring approach to permit inference of putative associations and provision of new intuitive visualizations. Overall, Open Targets is committed to a program of work that we believe will have a transformational effect on the science of therapeutic target validation including generating new experimental data. As the project proceeds we expect that new data generated from these experimental projects plus data from others will be fed into the Target Validation Platform, enriching it, and eventually providing new hypotheses for experimentation. The availability of integrated pre-competitive target validation data will help to enable new drug discovery programs to start with greater hypothesis support, and allow earlier termination of poorly supported programs.
